# The Combined Escherichia coli Nissle 1917 and Tryptophan Treatment Modulates Immune and Metabolome Responses to Human Rotavirus Infection in a Human Infant Fecal Microbiota-Transplanted Malnourished Gnotobiotic Pig Model

**DOI:** 10.1128/msphere.00270-22

**Published:** 2022-09-08

**Authors:** Husheem Michael, Vishal Srivastava, Loic Deblais, Joshua O. Amimo, Juliet Chepngeno, Linda J. Saif, Gireesh Rajashekara, Anastasia N. Vlasova

**Affiliations:** a Center for Food Animal Health, Department of Animal Sciences, Ohio Agricultural Research and Development Center, The Ohio State University, Wooster, Ohio, USA; b Department of Animal Production, Faculty of Veterinary Medicine, University of Nairobi, Nairobi, Kenya; University of Michigan-Ann Arbor

**Keywords:** *Escherichia coli* Nissle 1917, tryptophan, human rotavirus infection, metabolomics, lipidomics, neonatal gnotobiotic pigs, malnutrition

## Abstract

Human rotavirus (HRV) is a major cause of childhood diarrhea in developing countries where widespread malnutrition contributes to the decreased oral vaccine efficacy and increased prevalence of other enteric infections, which are major concerns for global health. Neonatal gnotobiotic (Gn) piglets closely resemble human infants in their anatomy, physiology, and outbred status, providing a unique model to investigate malnutrition, supplementations, and HRV infection. To understand the molecular signatures associated with immune enhancement and reduced diarrheal severity by Escherichia coli Nissle 1917 (EcN) and tryptophan (TRP), immunological responses and global nontargeted metabolomics and lipidomics approaches were investigated on the plasma and fecal contents of malnourished pigs transplanted with human infant fecal microbiota and infected with virulent (Vir) HRV. Overall, EcN + TRP combined (rather than individual supplement action) promoted greater and balanced immunoregulatory/immunostimulatory responses associated with greater protection against HRV infection and disease in malnourished humanized piglets. Moreover, EcN + TRP treatment upregulated the production of several metabolites with immunoregulatory/immunostimulatory properties: amino acids (*N*-acetylserotonin, methylacetoacetyl-CoA), lipids (gamma-butyrobetaine, eicosanoids, cholesterol-sulfate, sphinganine/phytosphingosine, leukotriene), organic compound (biliverdin), benzenoids (gentisic acid, aminobenzoic acid), and nucleotides (hypoxathine/inosine/xanthine, cytidine-5′-monophosphate). Additionally, the levels of several proinflammatory metabolites of organic compounds (adenosylhomocysteine, phenylacetylglycine, urobilinogen/coproporphyrinogen) and amino acid (phenylalanine) were reduced following EcN + TRP treatment. These results suggest that the EcN + TRP effects on reducing HRV diarrhea in neonatal Gn pigs were at least in part due to altered metabolites, those involved in lipid, amino acid, benzenoids, organic compounds, and nucleotide metabolism. Identification of these important mechanisms of EcN/TRP prevention of HRV diarrhea provides novel targets for therapeutics development.

**IMPORTANCE** Human rotavirus (HRV) is the most common cause of viral gastroenteritis in children, especially in developing countries, where the efficacy of oral HRV vaccines is reduced. Escherichia coli Nissle 1917 (EcN) is used to treat enteric infections and ulcerative colitis while tryptophan (TRP) is a biomarker of malnutrition, and its supplementation can alleviate intestinal inflammation and normalize intestinal microbiota in malnourished hosts. Supplementation of EcN + TRP to malnourished humanized gnotobiotic piglets enhanced immune responses and resulted in greater protection against HRV infection and diarrhea. Moreover, EcN + TRP supplementation increased the levels of immunoregulatory/immunostimulatory metabolites while decreasing the production of proinflammatory metabolites in plasma and fecal samples. Profiling of immunoregulatory and proinflammatory biomarkers associated with HRV perturbations will aid in the identification of treatments against HRV and other enteric diseases in malnourished children.

## INTRODUCTION

Human rotavirus (HRV) is a leading cause of diarrhea in children. It is associated with significant morbidity and mortality, especially in developing countries (1). Coincidently, the efficacy of RV vaccine is low in Low and Middle Income Countires (LMICs) (2). Malnutrition, micronutrient deficiencies, and interference by maternal milk antibodies are implicated in the lower efficacy of oral RV vaccines (3–5). Probiotics are increasingly used to enhance oral vaccine responses and treat enteric infections (6) and ulcerative colitis in children (7). The probiotic Escherichia coli Nissle 1917 (EcN) has been widely used in the treatment of ulcerative colitis in humans (8). EcN lacks virulence factors and possesses unique health-promoting properties (9). Our research group has previously shown that EcN protects gnotobiotic (Gn) pigs against HRV infection and decreases the severity of diarrhea by modulating innate and adaptive immunity and protecting the intestinal epithelium by binding HRV via histo-blood group antigen-like bacterial glycans (10–12).

Tryptophan (TRP) is one of the essential amino acids of protein metabolism, and the kynurenine-to-tryptophan ratio is negatively associated with the linear growth of children (13, 14). TRP also plays an important role in immune regulation. Altered TRP homeostasis coinciding with decreased serum angiotensin I-converting enzyme 2 levels has been observed previously in association with virulent (Vir) HRV challenge of Gn pigs fed a protein-calorie-deficient diet (15). TRP supplementation alleviated intestinal inflammation and normalized intestinal microbiota in malnourished mice (16). Moreover, it is catabolized via the indoleamine 2,3-dioxygenase pathway that has immunoregulatory effects, and its depletion has been well studied in the context of chronic infections (17). TRP is the precursor to serotonin, and EcN enhances the bioavailability of serotonin in gut tissues through modulation of its synthesis and clearance (18). Indole produced by EcN via tryptophanase also improves intestinal barrier function (19). Thus, EcN may influence and be affected by host nutritional status, and its coadministration with TRP may provide additional benefits to counteract malnutrition impacts efficiently and alleviate HRV disease. In addition, the interactions between EcN and TRP and the combined impact of these interactions on immune responses/metabolic activities to HRV disease in the context of host malnutrition have not been investigated previously.

Neonatal Gn pigs resemble infants in their physiology, anatomy, mucosal immune system, and outbred status (20–22). Gn pigs are immunocompetent at birth but immunologically immature (23). HRV-infected Gn pigs exhibit diarrhea, transient viremia, and intestinal lesions mimicking those in children (22). Gn pigs are caesarian derived and housed in sterile isolators to ensure their germfree status, permitting studies of gut colonization with single probiotic bacteria or complete fecal microbiota. Thus, Gn pigs are a unique animal model to study the impact of specific probiotics on host metabolism, the neonatal immune system, enteric viral infections, or oral vaccines without other confounding microbiota (24, 25). Importantly, transplantation of human infant fecal microbiota (HIFM) into Gn piglets recapitulates the infant microbial community (26, 27). The resulting microbiota-humanized Gn pigs allow manipulation of multiple variables and sampling of gut responses that are not possible in infants or conventional animal models.

Metabolomics is the analysis of the metabolites present in mammalian specimens in various states of health and disease and can identify disease biomarkers. All the biological samples can be investigated, but plasma, serum, urine, and feces are used commonly because the fecal metabolome reflects the host-microbiota interactions, in contrast to plasma/serum, which more closely captures the host’s metabolism. Depending on the type of biological material tested, various metabolites can be discovered (28). Recent findings confirmed alterations in the numbers of metabolites, mainly related to lipids and amino acids, in the serum of patients with inflammatory bowel disease (29–31).

Our study aimed to determine the usefulness of global nontargeted metabolomics and lipidomics of plasma and fecal metabolites in a humanized malnourished Gn pig model supplemented with EcN and TRP. Further, we investigated the potential immunoregulatory and proinflammatory biomarkers associated with reduced HRV severity in children. Additionally we evaluated the association between metabolites, immune responses, and HRV disease severity.

We hypothesized that combined EcN and TRP will enhance host immune responses and alter host metabolites in the humanized HIFM Gn pigs, which may be associated with moderating HRV infection. We further hypothesized that the identification of HRV-associated metabolic biomarkers will delineate host pathways and molecular targets, which can be used to develop HRV therapeutics targeting pathways perturbed by HRV. Our preliminary studies have indicated that treatment of malnourished humanized Gn pigs with combined EcN + TRP ameliorated the HRV diarrhea rather than single (EcN or TRP) supplementation.

## RESULTS

### EcN and TRP combination increased survival rate and body weight, reduced viral shedding and diarrheal occurrence, and enhanced immunological responses.

**FIG 1:**
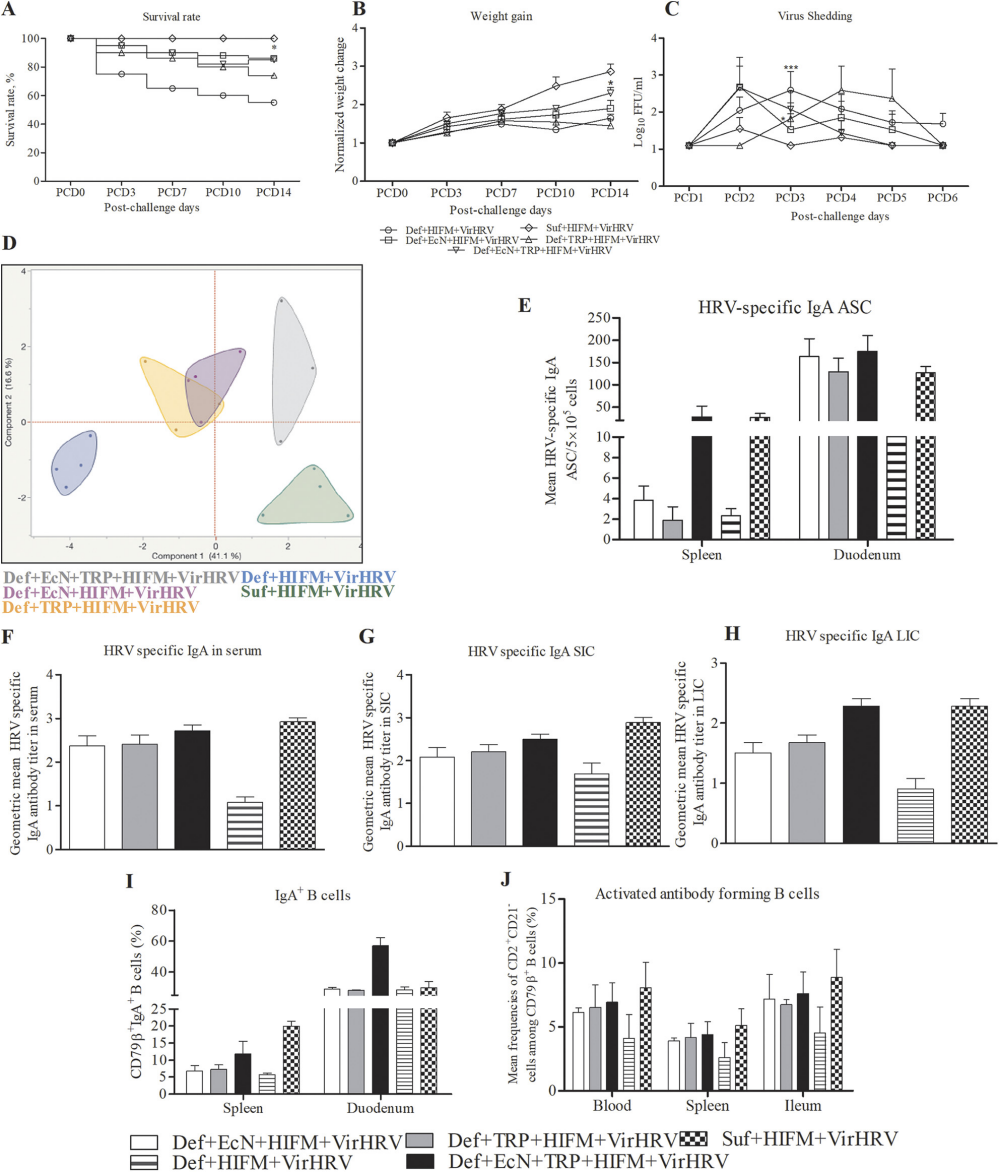
EcN + TRP treatment enhanced B cell immune responses post-VirHRV challenge. Survival rate (A) and normalized weight gain of pigs (B) after HIFM transplantation, supplementation, and VirHRV challenge were monitored for 2 weeks. (C) Virus shedding was determined by cell culture immunofluorescence assay and expressed as log_10_ FFU/ml. (D) Principal-component analysis (PCA) of immunological parameters. (E) Mean HRV-specific IgA antibody-secreting cells (ASCs) in splenic and duodenal cells. (F to H) Geometric mean titers (log_10_) HRV-specific IgA antibody in serum, small intestinal contents (SIC), and large intestinal content (LIC). (I) Mean frequencies of CD79β^+^IgA^+^ B cells in splenic and duodenal cells. (J) Mean frequencies of activated antibody-forming (CD79β^+^CD21^+^CD2^−^) B cells in systemic and ileal cells. Data are shown as means ± SEM, and data are compared with untreated group pigs. Gnotobiotic (Gn) pigs were cesarian derived and transplanted with HIFM at 4 days of age, post-HIFM transplantation day (PBTD) 0. Pigs were fed a deficient (Def) and/or sufficient (Suf) diet, supplemented orally with Escherichia Coli Nissle 1917 (EcN) and/or tryptophan (TRP) at PBTD7, subsequently challenged with virulent (Vir) HRV at PBTD9-10/postchallenge day (PCD) 0, and euthanized on PBTD24/PCD14.

EcN, TRP, and EcN + TRP supplementations increased the survival rates to 74%, 85%, and 86%, respectively, compared with the untreated (Def) pigs’ survival rate of 55% (Fig. 1A). During the first week of age, all the piglets had gained weight only marginally. At 2 weeks of age, a significant weight gain was evident in EcN + TRP pigs followed by EcN- and TRP-treated pigs compared with control untreated Def pigs (Fig. 1B). Analysis revealed that EcN + TRP-treated pigs had decreased peak virus shedding titers at PCD3 to PCD5 compared with other groups (Fig. 1C). Whereas single EcN or TRP treatment pigs had decreased peak virus shedding titers later at PCD5 to PCD6. Our results showed decreased diarrheal occurrence in EcN + TRP (28%) group compared with EcN (33%), TRP (42%), and Def group (75%). The TRP treatment group had the lowest diarrheal score (3.7) followed by EcN + TRP (4.1), EcN (5.1) treatments and control Def (8.0) groups (Table 1). Moreover, delayed onset of diarrhea was observed in EcN + TRP (4.0), EcN (3.0), and TRP (4.3) compared with Def (2.6) groups. Additionally, the shortened duration of diarrhea was also observed in EcN + TRP (0.3)-treated followed by EcN (1.0)- and TRP (0.4)-treated groups compared with Def (4.0) group pigs. Furthermore, diarrheal scores were correlated with viral shedding titers (*R* = 0.44, *P* = 0.02).

**TABLE 1:** Summary of diarrhea following VirHRV challenge (PCD1–PCD6)

Groups^*a*^	*n*	Diarrhea^*b*^ (%)	Mean cumulative fecal score^*c*^	Mean days to onset of diarrhea	Mean duration of diarrhea^*d*^
Def + EcN + HIFM + VirHRV	6	33	5.1*^e^	3.0	1.0
Def + TRP + HIFM + VirHRV	7	42	3.7*	4.3	0.4
Def + EcN + TRP + HIFM + VirHRV	7	28	4.1*	4.0	0.3
Def + HIFM + VirHRV	6	75	8.0**^f^	2.6	4.0
Suf + HIFM + VirHRV	7	57	6.7***^g^	3.0	1.0

a Gnotobiotic (Gn) pigs were transplanted with human infant fecal microbiota (HIFM) at 4 days of age, post-HIFM transplantation day (PBTD) 0. Pigs were fed either deficient (Def) or sufficient (Suf) diet. Pigs were supplemented orally with E. coli Nissle (EcN) 1917 and/or tryptophan (TRP) at PBTD7, subsequently challenged with virulent human rotavirus (VirHRV) at PBTD9-10/postchallenge day (PCD) 0 and pigs were euthanized on PBTD24/PCD14.

b Pigs with fecal score > 1 were considered diarrheic. Fecal consistency was scored as follows: 0, normal; 1, pasty; 2, semiliquid; and 3, liquid.

c Mean of total fecal score from PCD1 to PCD6. Means in the same column with different asterisks differ significantly (determined by two-way ANOVA followed by Bonferroni posttest, *P* < 0.05).

d Mean of the total days with fecal score > 1.

e *, *P* < 0.05.

f **, *P* < 0.01.

g ***, *P* < 0.001.

Principal-component analysis (PCA) identified immunological similarities/dissimilarities among the treatment groups (Fig. 1D). Clear immunological segregation was observed between Suf and Def diet pig groups expanding over 41% variance. Malnourished piglets supplemented with EcN + TRP and EcN had the highest immunological resemblance followed by TRP supplementation compared with the Suf pig group at component 1.

Analysis of B cells revealed that EcN + TRP, EcN, and TRP treatments had increased numbers of HRV-specific IgA antibody-secreting cells (ASCs) in splenic (negatively correlated with viral shedding titers, *R* = −0.5, *P* = 0.001) and duodenal cells (Fig. 1E). Coincident with decreased diarrheal score and increased HRV-specific ASCs, EcN + TRP, EcN, and TRP treatments increased HRV-specific IgA antibody titers in serum, small intestinal content (SIC), and large intestinal contents (LIC) (Fig. 1F to H). IgA antibody is a major functional component of the humoral adaptive immune system at mucosal sites (32) and the levels of HRV-specific IgA antibodies in pigs strongly correlate with protection against HRV infection (33–35). Similar to ASCs and IgA antibody titers, EcN + TRP, EcN, and TRP treatments also increased frequencies of IgA^+^ B lymphocytes among splenic and duodenal mononuclear cells (MNCs) (Fig. 1I). This is in agreement with previous studies where oral administration of probiotics increased the number of IgA^+^ B cells in the lamina propria (36, 37). EcN + TRP, EcN, and TRP treatments numerically increased frequencies of activated/primed (CD21^+^CD2^−^CD79β^+^) B cells among systemic and ileal MNCs (Fig. 1J). Taken together these results indicate that concurrent EcN + TRP treatment enhanced B cells in circulation and the gut of malnourished VirHRV-challenged HIFM-transplanted Gn pig model.

**FIG 2:**
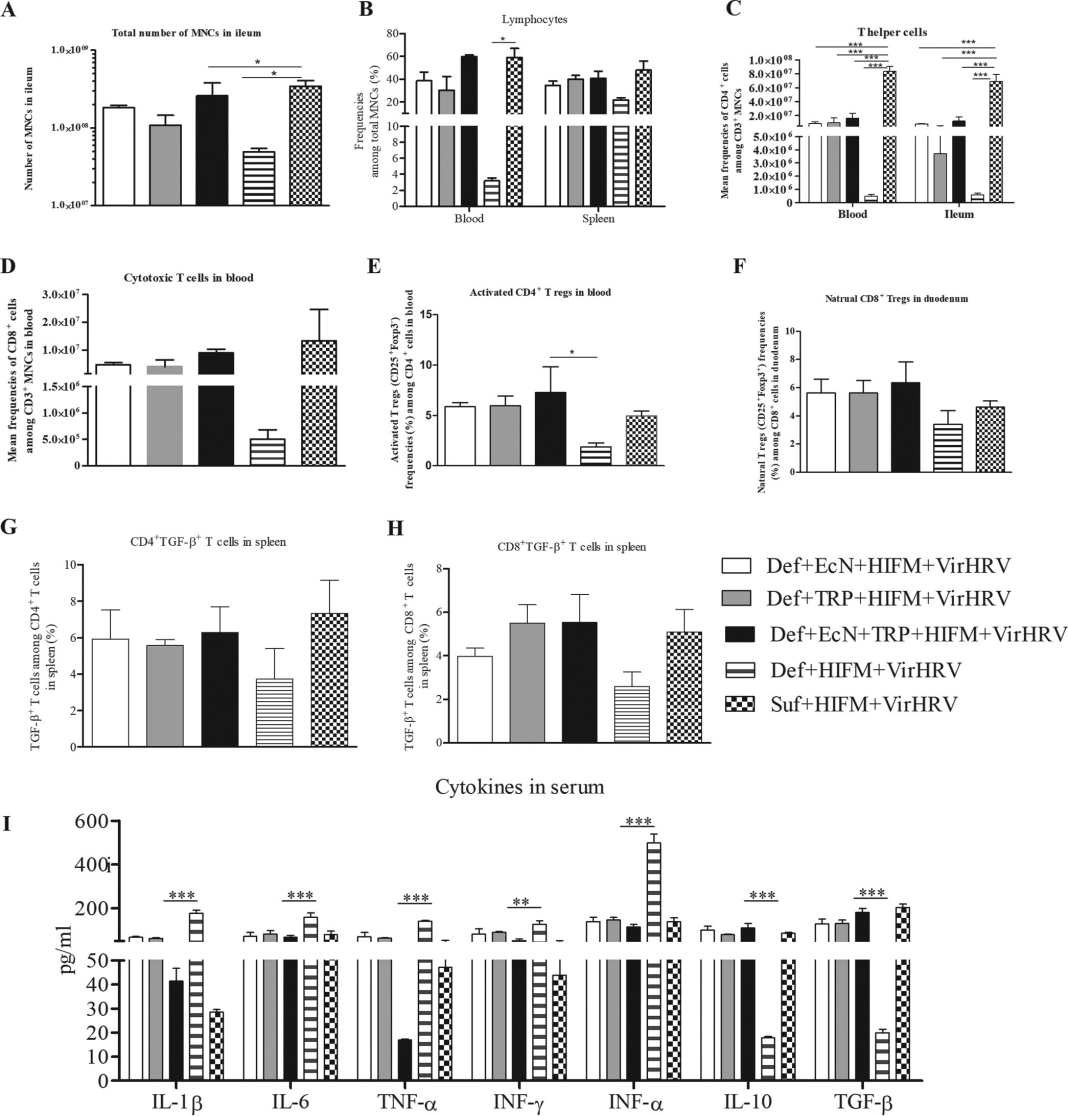
EcN + TRP treatment enhanced T cell immune responses post-VirHRV challenge. (A) Total numbers of mononuclear cells (MNCs) in the ileum. (B) Mean frequencies of lymphocytes among total MNCs in systemic tissues. (C) Mean frequencies of T-helper (CD3+CD4^+^) cells in the blood and ileal tissues. (D and E) Mean frequency of cytotoxic (CD3^+^CD8^+^) T and activated (CD4^+^CD25^+^FOXP3^−^) T-regulatory cells in blood cells. (F) Mean frequency of natural (CD8^+^CD25^+^FOXP3^+^) T-regulatory cells in duodenal tissues. (G and H) Mean frequency of CD4/CD8^+^TGF-β + T cells in splenic cells. (I) Cytokines profile was determined in serum samples collected at the terminal time point (PCD7). Data are shown as means ± SEM. Gnotobiotic (Gn) pigs were cesarian derived and transplanted with human infant fecal microbiota (HIFM) at 4 days of age, post-HIFM transplantation day (PBTD) 0. Pigs were fed a deficient (Def) and/or sufficient (Suf) diet, supplemented orally with Escherichia Coli Nissle 1917 (EcN) and/or tryptophan (TRP) at PBTD7, subsequently challenged with virulent (Vir) HRV at PBTD9-10/postchallenge day (PCD) 0, and euthanized on PBTD24/PCD14. *, *P* < 0.05; **, *P* < 0.01, and ***, *P* < 0.001.

Analysis of MNCs in different tissues revealed that EcN + TRP (significantly), EcN, and TRP treatments increased the total numbers of MNCs among ileal cells (Fig. 2A) and were negatively correlated with viral shedding titers (*R* = −0.5, *P* = 0.004). Similarly, EcN + TRP, EcN, and TRP treatments increased the frequencies of lymphocytes among total MNCs in systemic tissues (Fig. 2B). Furthermore, EcN + TRP, EcN, and TRP treatments significantly increased frequencies of T-helper (CD3^+^CD4^+^) cells among blood and ileal MNCs (Fig. 2C), and they were positively correlated with each other (*R* = 0.88, *P* = 0.0004). T-helper cell frequencies were positively correlated with total ileal MNC counts (*R* = 0.65, *P* = 0.007) and negatively correlated with viral shedding titers (*R* = −0.5, *P* = 0.01). Similarly, EcN + TRP (significantly), EcN, and TRP treatments increased the frequencies of cytotoxic (CD3^+^CD8^+^) T cells in blood (Fig. 2D); frequencies of activated (CD4^+^CD25^+^FoxP3^−^) Treg cells in blood (significantly, Fig. 2E) that correlated with HRV-specific IgA ASCs in duodenum (*R* = 0.88, *P* = 0.0004); and frequencies of natural cytotoxic (CD8^+^CD25^+^FoxP3^+)^ Treg cells among duodenal MNCs (Fig. 2F) that correlated with HRV-specific IgA ASCs in duodenal cells (*R* = 0.67, *P* = 0.006). Moreover, EcN + TRP, EcN, and TRP treatments increased the frequencies of CD4/CD8^+^ TGF-β + T cells among splenic MNCs (Fig. 2G and H). These results suggest that combined EcN + TRP treatment modulated the immunoregulatory environment and served as a potent inducer of intestinal immunity, restored gut immune homeostasis, and thus moderated HRV infection post-VirHRV challenge.

Additionally, proinflammatory and immunoregulatory cytokine responses post-VirHRV challenge were assessed by measuring levels of serum cytokines at terminal time points. Coincident with reduced diarrheal scores, EcN + TRP, EcN, and TRP treatments significantly reduced proinflammatory cytokine (interleukin-1β [IL-1β], IL-6, tumor necrosis factor-α [TNF-α], interferon-α [IFN-α], and IFN-γ) levels in serum, with combined EcN + TRP treatment showing remarkable reduction in IL-1β and TNF-α levels compared to single EcN/TRP treatments (Fig. 2I). Moreover, IFN-γ levels were correlated with diarrheal scores (*R* = 0.5, *P* = 0.003). These data suggest that concurrent EcN and TRP treatment reduced local (gut) inflammation caused by HRV infection. In contrast, immunoregulatory cytokine (IL-10 and TGF-β) levels were significantly increased in serum of EcN + TRP-, EcN-, and TRP-treated pigs (Fig. 2I), suggesting that EcN + TRP treatment induced an anti-inflammatory environment post-VirHRV challenge, thereby inhibiting proinflammatory cytokine responses.

**FIG 3:**
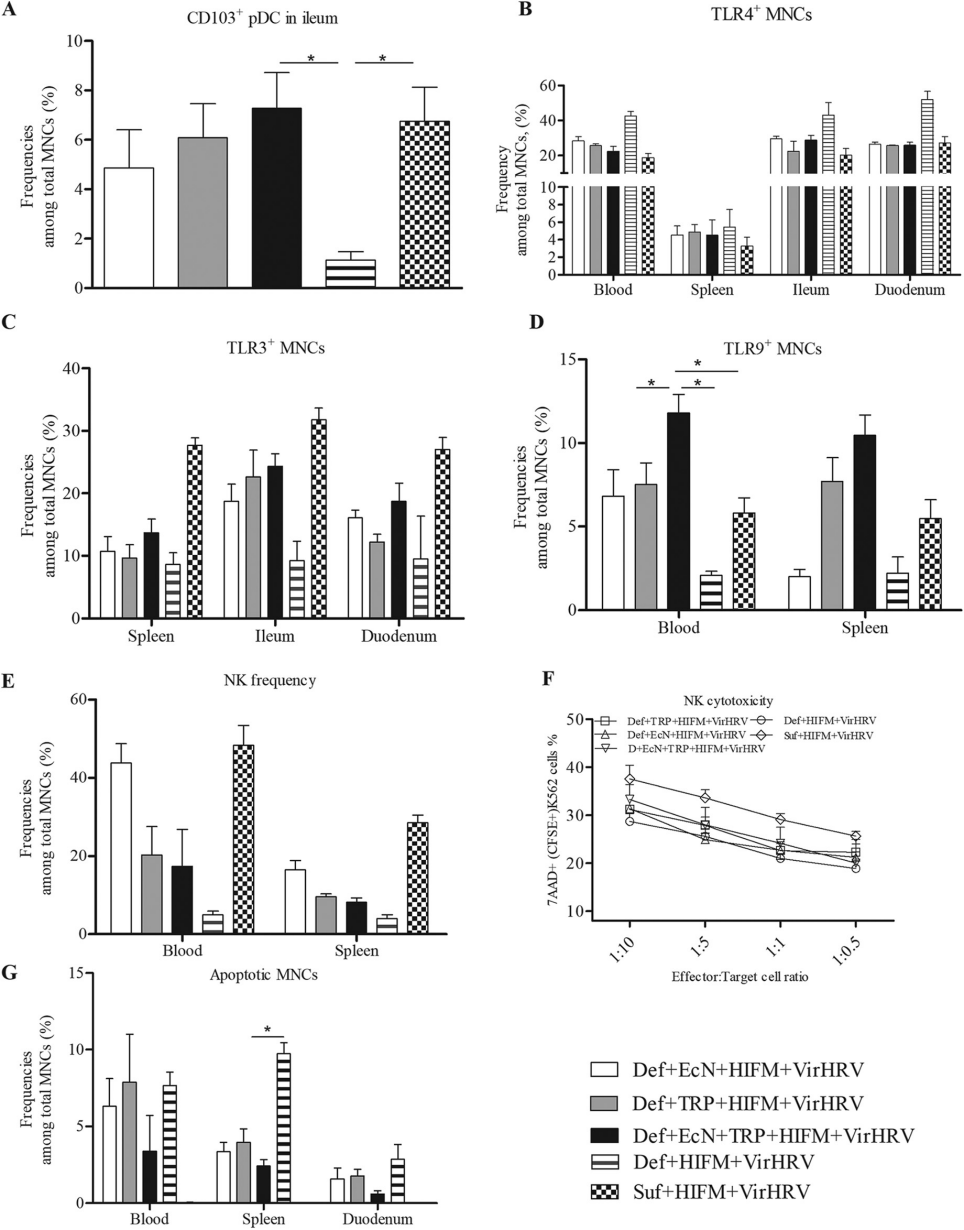
EcN + TRP treatment enhanced innate immune responses post-VirHRV challenge. (A) Mean frequency of CD103^+^ pDCs among total mononuclear cells (MNCs) in the ileum. (B) Mean frequencies of MNCs expressing TLR4 in systemic and intestinal tissues, (C) TLR3 in splenic and intestinal tissues, and (D) TLR9 in systemic tissues. (E) Mean frequency of NK (SWC3a^+^CD16^+^) and (F) NK cell function in systemic and blood MNCs. Blood MNCs and carboxyfluorescein diacetate succinimidyl ester (CFSE) stained K562 tumor cells were used as effector and target cells, respectively, and cocultured at set ratios to assess the NK cytotoxic function. For the effector, target cell cocultures were stained with 7-aminoactinomycin D (7AAD) after 12 h of incubation at 37°C, and the frequencies of CFSE-7AAD double-positive cells (lysed K562 target cells) were assessed by flow cytometry. (G) Mean frequencies of apoptotic MNCs (PI-/Annexin-APC-) among total blood MNCs. Data are shown as means ± SEM. Gnotobiotic (Gn) pigs were cesarian derived and transplanted with human infant fecal microbiota (HIFM) at 4 days of age, post-HIFM transplantation day (PBTD) 0. Pigs were fed a deficient (Def) and/or sufficient (Suf) diet, supplemented orally with Escherichia coli Nissle 1917 (EcN) and/or tryptophan (TRP) at PBTD7, subsequently challenged with VirHRV at PBTD9-10/postchallenge day (PCD) 0, and euthanized on PBTD24/PCD14. *, *P* < 0.05.

Analysis of innate immune responses demonstrated that EcN + TRP (significantly), EcN, and TRP treatments increased frequencies of CD103^+^ pDCs in ileal MNCs compared to the control Def group (Fig. 3A). Coincident with decreased diarrhea severity, the frequencies of Toll-like receptor 4 (TLR4) (associated with proinflammatory signaling) expressing MNCs were decreased (numerically) in systemic and intestinal MNCs of EcN + TRP-treated pigs compared with untreated Def pigs (Fig. 3B), which is consistent with previous observations (10, 38). The frequencies of TLR3 (associated with anti-RV protection) and TLR9 (associated with anti-inflammatory signaling) expressing MNCs were increased among intestinal, blood (significantly), and splenic MNCs, respectively, of EcN + TRP-, EcN-, and TRP-treated pigs compared with untreated Def pigs (Fig. 3C and D). Moreover, the numbers of TLR9 expressing MNCs in the spleen and blood were positively correlated with each other (*R* = 0.66 *P* = 0.007). Furthermore, frequencies of TLR9 expressing MNCs in blood were positively correlated with CD103^+^ pDCs in ileal MNCs (*R* = 0.67 *P* = 0.006) and were negatively correlated with diarrheal scores (*R* = −0.54, *P* = 0.021). These results indicate that enhanced TLR3/TLR9 expression associated with EcN + TRP treatment facilitated more efficient RV clearance. Similarly, EcN + TRP, EcN, and TRP treatments numerically increased the frequency of NK cells among systemic MNCs (Fig. 3E) and NK cell function in the blood MNCs (Fig. 3F). Moreover, compared with untreated Def pigs, EcN + TRP, EcN, and TRP treatments decreased the frequencies of apoptotic MNCs among blood, splenic and duodenal MNCs (Fig. 3G). Apoptotic MNCs in the blood was negatively correlated with CD103^+^ pDCs in ileum (*R* = −0.67, *P* = 0.006); lymphocytes in the blood (*R* = −0.71, *P* = 0.002); and T-helper cells in the blood (*R* = −0.66, *P* = 0.007). Additionally, apoptotic MNCs in blood and spleen were negatively correlated with viral shedding titers (*R* = −0.5 *P* = 0.02, *R* = −0.5 *P* = 0.04, respectively). These data suggest that EcN and TRP treatment either singly or in combination enhanced innate immune responses and improved protection against HRV infection associated with malnourishment and VirHRV in Gn pig model, with combined treatment (EcN + TRP) generally showing enhanced effects.

### Untargeted plasma and fecal metabolomics revealed differentially altered metabolites among EcN, TRP, and EcN + TRP treatment groups.

**FIG 4:**
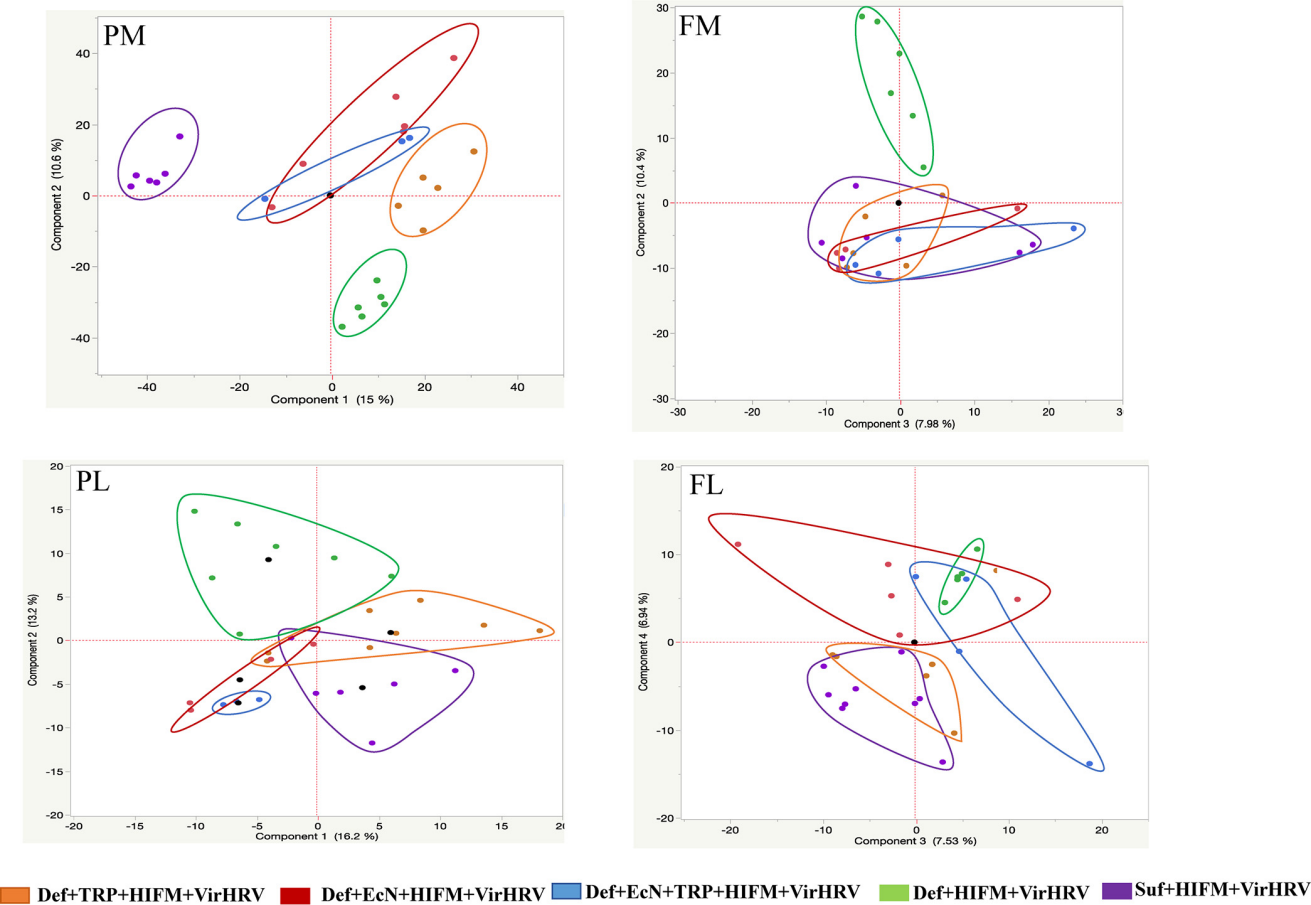
Principal-component analysis (PCA) of plasma/fecal metabolomics and lipidomics. Gnotobiotic (Gn) pigs were cesarian-derived and transplanted with human infant fecal microbiota (HIFM) at 4 days of age, post-HIFM transplantation day (PBTD) 0. Pigs were fed a deficient (Def) and/or sufficient (Suf) diet, supplemented orally with Escherichia coli Nissle 1917 (EcN) and/or tryptophan (TRP) at PBTD7, subsequently challenged with VirHRV at PBTD9-10/postchallenge day (PCD) 0, and euthanized on PBTD24/PCD14. PM, plasma metabolomics; PL, plasma lipidomics; FM, fecal metabolomics; FL, fecal lipidomics.

**FIG 5:**
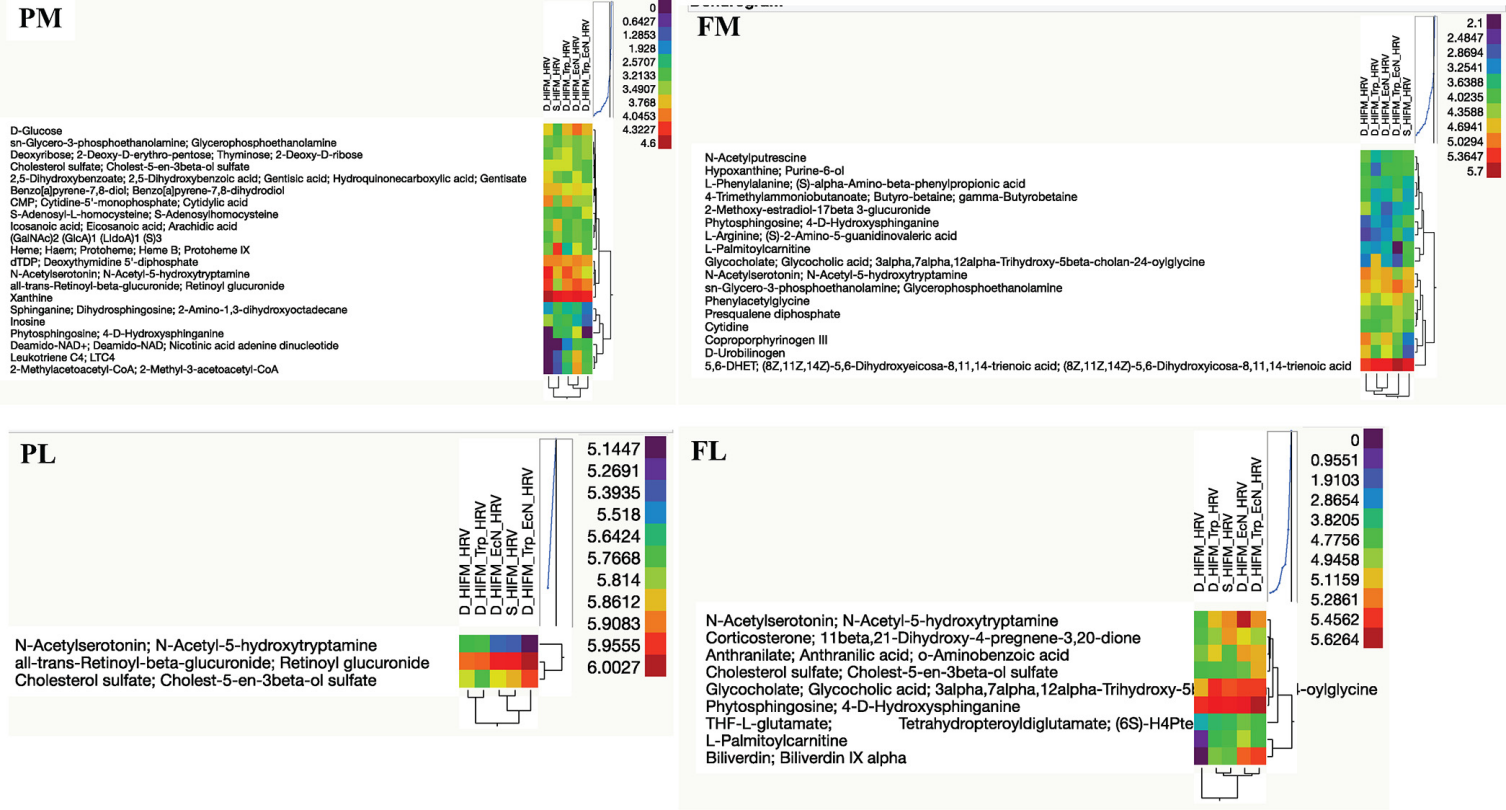
Heatmap of significant metabolites of plasma/fecal metabolomics and lipidomics. Heatmap visualizes the intensity/view of metabolites. More intense color represents the higher magnitude while the less intense color represents the lower magnitude of metabolite. In addition, the magnitude of metabolite is arranged to show their relatedness in the cluster heatmap. Gnotobiotic (Gn) pigs were cesarian derived and transplanted with human infant fecal microbiota (HIFM) at 4 days of age, post-HIFM transplantation day (PBTD) 0. Pigs were fed a deficient (Def) and/or sufficient (Suf) diet, supplemented orally with Escherichia coli Nissle 1917 (EcN) and/or tryptophan (TRP) at PBTD7, and, subsequently challenged with VirHRV at PBTD9-10/postchallenge day (PCD) 0, and euthanized on PBTD24/PCD14. PM, plasma metabolomics; PL, plasma lipidomics; FM, fecal metabolomics; FL, fecal lipidomics.

A total of 14,380 putative metabolites were detected in plasma metabolomics (PM); however, only 21 metabolites were found significant (*P* < 0.005) (Table 2 and 3 and Table S1 in the supplemental material). Multivariate analysis by principal-component analysis (PCA) and a heatmap revealed segregation of the treatment groups compared with the untreated (Def and Suf) groups (Fig. 4 and 5). The PCA plot identified and segregated metabolites among treated/untreated groups in PM samples. A close resemblance of metabolites was observed between EcN + TRP and EcN groups followed by the TRP group compared to untreated Suf diet group. Clear segregation of metabolites from the Def diet group of pigs revealed over 10% variance at component 2.

**TABLE 2:** Altered metabolites in different treatment groups

Treatment groups^*a*^	Immunoregulatory metabolites (↑)^*b*^	Proinflammatory metabolites (↓)^*c*^
Def + EcN + TRP + HIFM + VirHRV	Acetylserotonin, aminobenzoic acid, arginine, γ-butyrobetaine, biliverdin, cytidine-5′-monophosphate, cytidine, deoxythymidine, eicosanoids glycerophosethanolamine, hypoxanthine, inosine, leukotriene, phytosphingosine, presqualene diphosphate, retinoyl glucuronide, sphinganine, tetrahydropteroyldiglutamate (folic acid), trienoic acid, xanthine,	Acetylputrescine, adenosylhomocysteine, coproporphyrinogen, phenylalanine, phenylacetylglycine, urobilinogen
Def + EcN + HIFM + VirHRV	Acetylserotonin, acetyl glucosamine, gentisic acid/glycocholate/glycocholic acid, heme, methylacetoacetyl-CoA	Coproporphyrinogen, phenylalanine, urobilinogen
Def + TRP + HIFM + VirHRV	Nicotinamide adenine dinucleotide, palmitoylcarnitine, retinoyl glucuronide	Coproporphyrinogen, phenylalanine, urobilinogen

a Gnotobiotic (Gn) pigs were transplanted with human infant fecal microbiota (HIFM) at 4 days of age, post-HIFM transplantation day (PBTD) 0. Pigs were fed a deficient (Def) and/or sufficient (Suf) diet. Pigs were supplemented orally with E. coli Nissle (EcN) 1917 and/or tryptophan (TRP) at PBTD7, subsequently challenged with virulent human rotavirus (VirHRV) at PBTD9-10/postchallenge day (PCD) 0, and euthanized on PBTD24/PCD14.

b ↑ Indicates upregulated.

c ↓ Indicates downregulated.

**TABLE 3:** Dysregulated metabolites associated with different treatments in plasma metabolomics^*a*^

Metabolite	Pathway	Classification	Def + TRP + HIFM + HRV	Def + EcN + HIFM + HRV	Def + EcN + TRP + HIFM + HRV	Suf + HIFM + HRV
**d-glucose**	Glycolysis, gluconeogenesis,	Carbohydrates	−0.9	−0.4	−0.8	5.5
Deoxyribose	Pentose phosphate pathway	Carbohydrate	−0.5	−0.9	−0.7	1.7
***S*-adenosylhomocysteine; *S*-adenosyl-l-homocysteine**	Cysteine and methionine metabolism, biosynthesis of amino acids	Organic compound	1.2	1.8	−0.5	2.9
Heme; protoheme; heme B; protoheme IX	Biosynthesis of secondary metabolites, ABC transporters, ferroptosis, mineral absorption	Organic compound	8.7	−06	1.3	−0.1
**(GalNAc)2 (GlcA)1 (LIdoA)1** (S)**3**	Glycosaminoglycan degradation	Organic compound	1.4	−0.8	1.0	−0.6
**Cytidine-5′-monophosphate; CMP; cytidylic acid**	Pyrimidine metabolism	Nucleotide	1.3	3.4	2.9	4.1
**Nicotinic acid adenine dinucleotide**	Nicotinate and nicotinamide metabolism	Nucleotide	87	1,596	690	0
**Inosine**	Purine metabolism	Nucleotide	2.8	15	81.5	6.8
Deoxythymidine 5′-diphosphate	Pyrimidine metabolism	Nucleotide	1.4	1.0	1.3	1.0
**Xanthine**	Purine metabolism	Nucleotide	1.5	1.2	1.6	1.4
**Gentisic acid**	Benzoate degradation	Benzenoids	2.7	1.2	2.5	2.0
Benzo[a]pyrene-7,8-diol	Metabolism of xenobiotics by cytochrome P450	Benzenoids	1.4	1.1	1.3	1.0
**2-Methylacetoacetyl-CoA**	Valine, leucine, and isoleucine degradation	Amino acid	445	8,802	2,156	13
***N*-acetylserotonin; *N*-acetyl-5-hydroxytryptamine**	Tryptophan metabolism	Amino acid	1.5	3.1	5.2	3.9
**Sphinganine**	Sphingolipid metabolism	Lipid	−0.5	−0.4	2.0	−0.1
**Glycerophosphoethanolamine; sn-glycero-3-phosphoethanolamine**	Glycerophospholipid metabolism, ether lipid metabolism	Lipid	−0.7	1.1	−0.9	1.2
**Leukotriene C4**	Arachidonic acid metabolism, asthma	Lipid	1,644	6,654	1,517	18
**Eicosanoic acid; icosanoic acid; arachidic acid**	Biosynthesis of unsaturated fatty acids	Lipid	2.4	1.0	1.9	-0.4
**Retinoyl glucuronide;all-trans-retinoyl-beta-glucuronide**	Retinol metabolism	Lipid	2.0	1.3	2.0	6.3
Phytosphingosine; 4-d-Hydroxysphinganine	Sphingolipid metabolism	Lipid	549	3,931	1,020	645
Cholesterol sulfate; Cholest-5-en-3beta-ol sulfate	Steroid hormone biosynthesis	Lipid	1.2	1.8	2.1	1.0

a Gnotobiotic (Gn) pigs were transplanted with human infant fecal microbiota (HIFM) at 4 days of age, post-HIFM transplantation day (PBTD) 0. Pigs were fed deficient (Def) and/or sufficient (Suf) diets. Pigs were supplemented orally with E. coli Nissle (EcN) 1917 and/or tryptophan (TRP) at PBTD7, subsequently challenged with virulent human rotavirus (VirHRV) at PBTD9-10/postchallenge day (PCD) 0, and euthanized on PBTD24/PCD14. Treatment groups were compared with untreated (Def) control group pigs and presented as fold change. A negative sign represents decreased relative intensity. Metabolites highlighted in bold are correlated with immunological assays, determined by Spearman’s nonparametric correlation method.

10.1128/msphere.00270-22.2 TABLE S1 Excel file, relative intensities of dysregulated metabolites. Download Table S1, XLSX file, 0.02 MB. Copyright © 2022 Michael et al. 2022 Michael et al. https://creativecommons.org/licenses/by/4.0/ This content is distributed under the terms of the Creative Commons Attribution 4.0 International license.

A decreased concentration (based on relative intensity) of d-glucose and deoxyribose was observed among all treatment groups compared with untreated Def group (Table 3). Single EcN treatment increased the intensity of the following metabolites: *S*-adenosyl-l-homocysteine, cytidine-5′-monophosphate, nicotinic acid adenine dinucleotide, glycerophosphoethanolamine, leukotriene C4, 2-Methylacetoacetyl-CoA, and phytosphingosine. While single TRP treatment increased the relative intensity of heme, deoxythymidine 5′-diphosphate, gentisic acid, eicosanoic acid, benzo[a]pyrene-7,8-diol/benzo[a]pyrene-7,8-dihydrodiol, and (GalNAc)2 (GlcA)1 (LIdoA)1 (S).

The combined EcN + TRP treatment increased the relative intensity of inosine, xanthine, sphinganine, *N*-acetylserotonin/*N*-acetyl-5-hydroxytryptamine, and cholesterol sulfate. Interestingly, TRP and EcN + TRP treatment equally increased the relative intensity of retinoyl glycuronide.

A total of 4,765 putative metabolites were detected in fecal metabolomics (FM) analysis; however, only 17 metabolites were found significant (*P* < 0.005) (Table 4). Multivariate analysis by PCA plot and a heatmap revealed segregation of the treatment groups compared with the untreated (Def) group in FM analysis (Fig. 4 and 5 and Table S1). PCA identified metabolites among treatments/untreated groups in FM samples. Clear discrimination of metabolites was observed in Def pigs revealing over 10% variance at component 2. Malnourished piglets (Def group) supplemented with EcN + TRP, EcN, and TRP had the highest resemblance of metabolites with untreated Suf diet group pigs with over 8% variance at component 3.

**TABLE 4:** Dysregulated metabolites in fecal metabolomics^*a*^

Metabolite	Pathway	Classification	Def + TRP + HIFM + VirHRV	Def + EcN + HIFM + VirHRV	Def + EcN + TRP + HIFM + VirHRV	Suf + HIFM + VirHRV
*N*-acetylputrescine	Arginine and proline metabolism	Organic compound	−0.2	−0.3	−0.5	−0.9
**Coproporphyrinogen III**	Porphyrin and chlorophyll metabolism	Organic compound	−0.2	−0.5	−0.1	−0.1
**d-urobilinogen**	Biosynthesis of secondary metabolites	Organic compound	−0.1	−0.6	−0.2	−0.1
**Phenylacetylglycine**	Phenylalanine metabolism	Organic compound	−0.6	−0.8	1.4	−0.6
Cytidine	Pyrimidine metabolism	Nucleotide	−0.7	1.5	2.3	1.1
**Hypoxanthine; urine-6-ol**	Purine metabolism	Nucleotide	−0.1	1.0	1.0	1.2
**l-phenylalanine**	Phenylalanine	Amino acid	−0.4	−0.3	−0.5	−0.2
l-arginine; (*S*)-2-amino-5-guanidinovaleric acid	Arginine biosynthesis, biosynthesis of amino acids	Amino acid	3.6	5.0	27	7.2
***N*-acetylserotonin; *N*-acetyl-5-hydroxytryptamine**	Tryptophan metabolism	Amino acid	−0.5	−0.6	−0.1	−0.5
**Phytosphingosine; 4-d-hydroxysphinganine**	Sphingolipid metabolism	Lipid	2.2	1.6	3.5	11
5,6-DHET; (8Z,11Z,14Z)-5,6-dihydroxyeicosa-8,11,14-trienoic acid; (8Z,11Z,14Z)-5,6-	Arachidonic acid metabolism	Lipid	1.6	1.4	2.6	1.8
l-palmitoylcarnitine	Fatty acid degradation and metabolism	Lipid	−0.9	1.3	−0.4	4.6
Glycocholate; glycocholic acid;	Primary and secondary bile acid biosynthesis	Lipid	30	1.5	−0.4	5.2
2-Methoxy-estradiol-17beta 3-glucuronide	Steroid hormone biosynthesis	Lipid	−0.1	−0.4	−0.1	−0.1
Glycerophosphoethanolamine	Glycerophospholipid metabolism, lipid metabolism	Lipid	−0.5	1.0	2.0	−0.9
**Gamma-butyrobetaine**	Lysine degradation	Lipid	−0.3	−0.4	2.1	−0.3
Presqualene diphosphate	Sesquiterpenoid and triterpenoid biosynthesis	Lipid	−0.6	−0.6	1.1	1.0

a Gnotobiotic (Gn) pigs were transplanted with human infant fecal microbiota (HIFM) at 4 days of age, post-HIFM transplantation day (PBTD) 0. Pigs were fed deficient (Def) and/or sufficient (Suf) diet. Pigs were supplemented orally with E. coli Nissle (EcN) 1917 and/or tryptophan (TRP) at PBTD7, subsequently challenged with virulent human rotavirus (VirHRV) at PBTD9-10/postchallenge day (PCD) 0, and euthanized on PBTD24/PCD14. Treatment groups were compared with untreated (Def) group pigs and presented as fold change. A negative sign represents decreased relative intensity. Metabolites highlighted in bold are correlated with immunological assays, determined by Spearman’s nonparametric correlation method.

A decreased relative intensity of metabolites was observed among all treated groups compared with the untreated Def pigs, including *N*-acetylputrescine, l-phenylalanine, *N*-acetylserotonin/*N*-acetyl-5-hydroxytryptamine, 2-methoxy-estradiol-17beta 3-glucuronide, urobilinogen, and coproporphyrinogen III. However, combined EcN + TRP treatment also increased the relative intensity of the following metabolites glycerophosphoethanolamine; phenylacetylglycine; l-arginine; phytosphingosine; gamma-butyrobetaine; (8Z,11Z,14Z)-5,6-dihydroxyeicosa-8,11,14-trienoic acid; and presqualene diphosphate. Similarly, a single treatment of EcN increased the relative intensity of l-palmitoylcarnitine. Interestingly, EcN and EcN +TRP treatment equally increased the relative intensity of hypoxanthine/purine-6-ol.

Individual treatment with either EcN or TRP increased the relative intensity of phytosphingosine while combined EcN + TRP treatment profoundly increased the relative intensity of the same metabolite. A single treatment with EcN and TRP increased and decreased, respectively, while combined EcN + TRP treatment profoundly increased the relative intensity of cytidine. A single treatment with TRP profoundly increased the relative intensity of glycocholate/glycocholic acid. *N*-acetylserotonin, phytosphingosine, and glycerophosphoethanolamine metabolites were identified commonly in PM and FM samples.

### Untargeted plasma and fecal lipidomics revealed differentially altered metabolites among EcN, TRP, and EcN + TRP treatment groups.

A total of 1,084 putative metabolites were detected in plasma lipidomics (PL) analysis. Among those, only three metabolites were found to differ significantly (*P* < 0.005) (Table 5 and Table S1). Multivariate analysis by PCA plot and a heatmap revealed segregation of treatment groups from the untreated group in PL analysis (Fig. 4 and 5). PCA plot categorized metabolites among treatments/untreated groups in PL samples. Clear segregation of metabolites was observed for Def diet group pigs expanding over 13% variance at component 2. Malnourished piglets supplemented with EcN + TRP, EcN, and TRP groups had closely resembled metabolites with untreated Suf group pigs over 16% variance at component 1.

**TABLE 5:** Dysregulated metabolites in plasma lipidomics^*a*^

Metabolite	Pathway	Classification	Def + TRP + HIFM + VirHRV	Def + EcN + HIFM + VirHRV	Def + EcN + TRP + HIFM + VirHRV	Suf + HIFM + VirHRV
*N*-acetylserotonin; *N*-acetyl-5-hydroxytryptamine	Tryptophan metabolism	Amino acid	−0.9	1.7	3.6	1.9
**Retinoyl glucuronide; all-trans-retinoyl-beta-glucuronide**	All-trans-retinoyl-beta-glucuronide	Lipid	−0.9	−0.8	−0.8	−0.8
**Cholesterol sulfate; cholest-5-en-3beta-ol sulfate**	Steroid hormone biosynthesis	Lipid	1.1	−0.9	−0.7	−0.9

a Gnotobiotic (Gn) pigs were transplanted with human infant fecal microbiota (HIFM) at 4 days of age, post-HIFM transplantation day (PBTD) 0. Pigs were fed deficient (Def) and/or sufficient (Suf) diets. Pigs were supplemented orally with E. coli Nissle (EcN) 1917 and/or tryptophan (TRP) at PBTD7, subsequently challenged with virulent human rotavirus (VirHRV) at PBTD9-10/postchallenge day (PCD) 0, and euthanized on PBTD24/PCD14. Treatment groups were compared with untreated (Def) group pigs and presented as fold change. A negative sign represents the decreased relative intensity. Metabolites highlighted in bold are correlated with immunological assays, determined by Spearman’s nonparametric correlation method.

Analysis revealed that single TRP treatment increased the relative intensity of cholesterol sulfate compared to untreated (Def) group pigs. Moreover, EcN and EcN + TRP treatment increased the relative intensity of *N*-acetylserotonin/*N*-acetyl-5-hydroxytryptamine. Lastly, retinoyl glucuronide relative intensity was decreased among all treatment groups compared with the untreated group.

A total of 3,517 putative metabolites were detected in fecal lipidomics (FL) analysis. Among those, nine metabolites differed significantly (*P* < 0.005) (Table 6 and Table S1). Multivariate analysis by PCA plot and a heatmap revealed segregation of treatment groups compared with the untreated group in FL analysis (Fig. 4 and 5). PCA plot revealed that TRP-treated pig and Suf group metabolites were similar compared to the other treatment groups in FL samples. Untreated (Def) and other treatment group metabolites overlapped with each other.

**TABLE 6:** Dysregulated metabolites in fecal lipidomics^*a*^

Metabolite	Pathway	Classification	Def + TRP + HIFM + VirHRV	Def + EcN + HIFM + VirHRV	Def + EcN + TRP + HIFM + VirHRV	Suf + HIFM + VirHRV
THF-l-glutamate; tetrahydropteroyldiglutamate; (6S)-H4PteGlu2	Folate biosynthesis	Organic compound	3.8	36.4	12.7	5.5
**Biliverdin; biliverdin IX alpha**	Biosynthesis of secondary metabolites	Organic compound	79,714	222,806	263,611	71,308
**Anthranilate; anthranilic acid; *o*-aminobenzoic acid**	Aminobenzoate degradation	Benzenoids	1.5	1.9	1.7	0.8
***N*-acetylserotonin; *N*-acetyl-5-hydroxytryptamine**	Tryptophan metabolism	Amino acid	3.0	9.4	3.8	3.9
Corticosterone; 11beta,21-dihydroxy-4-pregnene-3,20-dione	Steroid hormone biosynthesis	Lipid	1.3	1.7	1.4	2.5
Phytosphingosine; 4-d-hydroxysphinganine	Sphingolipid metabolism	Lipid	1.1	1.2	1.4	1.1
Cholesterol sulfate; cholest-5-en-3beta-ol sulfate	Steroid hormone biosynthesis	Lipid	1.5	1.9	3.7	1.6
**l-palmitoylcarnitine**	Fatty acid degradation	Lipid	1,997	14,425	4,426	6,100
Glycocholate; glycocholic acid; 3alpha,7alpha,12alpha-trihydroxy-5beta-cholan-24-oylglycine	Primary and secondary bile acid biosynthesis, cholesterol metabolism, Bile secretion	Lipid	2.1	1.9	1.9	1.7

a Gnotobiotic (Gn) pigs were transplanted with human infant fecal microbiota (HIFM) at 4 days of age, post-HIFM transplantation day (PBTD) 0. Pigs were fed deficient (Def) and/or sufficient (Suf) diets. Pigs were supplemented orally with E. coli Nissle (EcN) 1917 and/or tryptophan (TRP) at PBTD7, subsequently challenged with virulent human rotavirus (VirHRV) at PBTD9-10/postchallenge day (PCD) 0, and euthanized on PBTD24/PCD14. Treatment groups were compared with untreated (Def) group pigs and presented as fold change. A negative sign represents the decreased relative intensity. Metabolites highlighted in bold are correlated with immunological assays, determined by Spearman’s nonparametric correlation method.

Analysis revealed that single EcN treatment increased the relative intensity of *N*-acetylserotonin/*N*-acetyl-5-hydroxytryptamine, corticosterone, tetrahydropteroyldiglutamate, l-palmitoylcarnitine, and aminobenzoic acid. Moreover, the analysis revealed that a single TRP treatment increased glycocholate/glycocholic acid. Finally, EcN + TRP treatment increased the relative intensity of phytosphingosine, cholesterol sulfate, and biliverdin.

Interestingly, in addition to the metabolites associated with lipids; the metabolites associated with amino acids, organic compounds, and benzenoids were also detected in the lipidomics analysis (Tables 5 and 6). *N*-acetylserotonin and cholesterol sulfate metabolites were identified in both PL and FL samples.

## DISCUSSION

The multifactorial pathobiology of malnutrition is associated with perturbed intestinal homeostasis, epithelial dysfunction, altered metabolism, impaired immunity, intestinal inflammation, and malabsorption (39, 40). Impaired immune defense compromises gut integrity and causes a microbial imbalance that can predispose malnourished hosts to intestinal infections (41). These concerns require further research to improve the condition and treatment options of malnourished hosts.

### EcN ± TRP supplementation enhanced immune responses.

Using a malnourished HIFM-transplanted Gn pig model of VirHRV infection, we evaluated the effects of treatment with EcN and/or TRP, and EcN + TRP on multiple aspects of the immune response and clinical parameters of the enteric disease. Our results suggested that supplementation have resulted in reduced diarrhea in HIFM piglets. Malnutrition-associated enteric dysfunction contributes to persistent systemic and intestinal inflammation that further exacerbates physiological and intestinal abnormalities, thus negatively affecting growth (39, 42, 43). In this study, a significant weight gain in EcN + TRP-treated pigs was observed compared to untreated Def pigs suggesting the restoration of intestinal homeostasis. In addition, EcN/TRP supplementation may have contributed to the energy absorption that led to increased survival rates. Concurrent EcN + TRP treatment increased systemic activated T regs that are associated with higher serum immunoregulatory cytokine levels. This could have contributed to reduced proinflammatory serum cytokine levels indicative of an anti-inflammatory microenvironment, thus reducing the severity of HRV disease (44–46). Moreover, enhanced immunoregulatory cytokines could have also contributed to increased HRV-specific IgA responses observed in EcN + TRP-treated pigs through TLR9 signaling (47–53). Interestingly, the relative intensity of deoxyribose was reduced (although not significantly) in treated Def pigs compared with Suf pigs in PM samples, as deoxyribose act as an agonist and activates TLR9 (54). In addition, increased serum levels of IFN-γ and TNF-α (an indicator of inflammation) in untreated malnourished pigs and reduced levels of these cytokines in EcN + TRP-, EcN-, and TRP-treated pigs are suggestive of moderation of inflammation (11, 12, 26, 55–57). We previously demonstrated that protein malnutrition altered innate immunity in HRV-infected neonatal Gn pigs (26). This study suggests that EcN + TRP supplementation (i) can inhibit the proapoptotic effects of HRV infection by inhibiting TLR-mediated proapoptotic signaling or by activating antiapoptotic pathways (10), and (ii) support the adequate adaptive immune function (38, 58) and programmed cell death by enhancing innate immune functions (53, 59–64).

Untargeted global metabolomics revealed alterations in both plasma and fecal metabolites and metabolic pathways that may have contributed to prophylactic diarrheal protection. Our results further highlight those metabolites/compounds that may be associated with protection against HRV infection related to increased production of gut-protective immunomodulatory, and anti-HRV metabolites/compounds. Moreover, several metabolites were correlated with immune responses (Table S2).

10.1128/msphere.00270-22.3 TABLE S2 Excel file, correlations of immune responses with significantly altered metabolites in plasma and fecal metabolomic and lipidomic analysis. Download Table S2, XLSX file, 0.01 MB. Copyright © 2022 Michael et al. 2022 Michael et al. https://creativecommons.org/licenses/by/4.0/ This content is distributed under the terms of the Creative Commons Attribution 4.0 International license.

### EcN ± TRP supplementation altered amino acid-associated metabolites.

TRP is an essential amino acid in protein metabolism and the precursor of serotonin. TRP homeostasis and catabolism are disrupted by a protein deficiency that affects adaptive immune responses in Gn pigs while TRP supplementation enhances the frequencies of T-regulatory cells in pigs (15). Moreover, it was demonstrated that malnourished hosts were unable to catabolize TRP indicating protein turnover and the TRP degradation pathway was suppressed (65). In our current study, *N*-acetylserotonin/*N*-acetyl-5-hydroxytryptamine (involved in TRP metabolism) intensity was increased in EcN (FL and EcN) + TRP (PM and PL)-treated malnourished pigs. These metabolites are involved in adaptive immune responses suggesting that EcN and TRP supplementation may have improved or restored amino acid and protein homeostasis, which coincided with enhanced immunological parameters. Methylacetoacetyl-CoA is an intermediate in the degradation of isoleucine. Dietary isoleucine improved the growth performance and immune function via activation of pattern recognition receptor signaling pathways in RV-infected piglets (66). In our study, methylacetoacetyl-CoA intensity was substantially increased in EcN (PM)-treated pigs, indicating the role of EcN in enhancing immune responses, and it may also lead to the weight gain of malnourished piglets. Phenylalanine is an essential amino acid and is implicated in several body functions. However, during infections or inflammatory conditions, phenylalanine is significantly increased in serum (67). In addition, phenylalanine is excreted in greater amounts by stunted children suggesting a functional modulation of the gut immune/microbiome due to increased proteolytic activity (68). Our data demonstrated that phenylalanine intensity was decreased among all treated pigs compared with untreated Def pigs in the FM analysis. This is indicative of immune modulation in the host gastrointestinal system as it correlates with enhanced immunological responses.

### EcN ± TRP supplementation altered benzenoid-associated metabolites.

Aminobenzoic acid belongs to the family of benzoic acid, and it is known to improve gut functions via regulating enzyme activity, redox status, immunity, and microbiota (69). In this study, EcN treatment increased aminobenzoic acid levels in FL. This suggests that EcN supplementation enhanced nutrient digestion, absorption, and barrier function of the malnourished piglets (11). Gentisic acid, a metabolite of aspirin, inhibits prostaglandin (involved in inflammatory responses) formation in response to LPS (70). Our data revealed that TRP treatment increased gentisic acid intensity in plasma. This suggests that TRP supplementation may have anti-inflammatory components, and thus, it modulates adaptive and innate immune responses and protects against HRV diarrhea in malnourished hosts.

### EcN ± TRP supplementation altered organic compound-associated metabolites.

The elevated intensity of homocysteine has been implicated in intestinal inflammation (71). Moreover, increased serum homocysteine levels in inflammatory bowel syndrome in rats led to intestinal epithelial barrier dysfunction (72). Adenosylhomocysteine is the biosynthetic precursor to homocysteine, a metabolic indicators of cellular methylation and a biomarker of cardiovascular disease. It is decreased in the EcN + TRP-treated pigs in PM indicating that the supplementation may have reduced oxidative stress, decreased inflammation, lowered permeability, and modulated the mucosal immune system in the intestinal tract of neonatal pigs (73, 74). EcN + TRP treatment modulates adenosylhomocysteine that may have favorably shaped the mucosal immune response and enteric system to protect enterocytes and gut microbiota from oxidative damage and maintain normal gut motility during HRV infection and diarrhea (11, 73–75). Moreover, reducing inflammation in the gastrointestinal tract may provide the host with improved barrier function (76, 77). Biliverdin, a product of heme catabolism, can act as a cytoprotective and anti-inflammatory molecule. Recent data have shown that biliverdin is involved in innate immune responses resulting from acute insult and injury (78). Malnutrition can cause a decreased intensity of heme oxygenase activity leading to decreased biliverdin intensity as observed in FL samples of untreated pigs. However, EcN + TRP treatment increased the levels of biliverdin in FL samples, indicating an improved innate immune response and nutritional status that may have led to an increased survival rate and weight gain in the malnourished pigs. Coproporphyrinogen III is an intermediate metabolite in the normal biosynthesis of heme. This compound is not normally produced by the human body in a normal state; however, it is produced during protein-calorie malnutrition (79). Our current results show that coproporphyrinogen levels were decreased in all treatment groups in FM analysis. We hypothesized that EcN ± TRP treatment increased the ability of the liver to convert coproporphyrin to protoporphyrin (which acts as a precursor to heme/hemoglobin) by enhancing the enzyme coproporphyrinogen oxidase. This in turn improved adaptive and innate immune responses and nutritional status of the malnourished piglets and thus decreased diarrhea and virus shedding. Urobilinogen is a colorless by-product of bilirubin reduction, and increased levels are implicated in several liver diseases. Previous studies have shown that malnutrition induces gut atrophy and impaired small intestinal epithelial barrier in malnourished pigs, which may have resulted in the elevation of bilirubin (26, 80). Our current data demonstrate that all treatment groups had decreased urobilinogen levels in FM relative to the untreated Def group, indicating that the treatments may have protected the intestinal epithelium maintaining the absorptive function and thus decreasing the severity of diarrhea in HRV infection (11). These findings are in agreement with previous studies that EcN/TRP supplementation enhanced the immune responses in malnourished pigs (15, 48). Phenylacetylglycine is a putative biomarker of phospholipidosis, and its metabolic activity is disrupted by malnutrition (68), while EcN + TRP enhanced its levels in FM, indicative of enhanced adaptive and innate immune function.

### EcN ± TRP supplementation altered lipid-associated metabolites.

Sphingolipids show the potential to impact chronic inflammatory diseases through inhibiting intestinal lipid absorption, altering the gut microbiota, activation of anti-inflammatory nuclear receptors, and LPS neutralization (81). Our current data show that EcN + TRP treatment increased the sphinganine levels in PM analysis, thus indicating improved adaptive and innate immune responses. Phytosphingosine exhibits anti-inflammation and antimicrobial activities; in addition, it stimulated epidermal differentiation through PPAR activation (82). Similarly, like other sphingolipids, EcN + TRP treatment increased phytosphingosine intensity in FM and FL while EcN treatment increased it in PM analysis. This suggests EcN + TRP and/or TRP enhanced lipid metabolism, thus ameliorating HRV infection. Arachidonic acid is a precursor of inflammatory mediators and dysregulated during protein-calorie malnutrition due to perturbation of essential fatty acid metabolism (83). In this study, EcN treatment increased leukotriene (a metabolite of arachidonic acid) intensity relative to untreated Def pig (indicating an impaired immune response) in PM analysis suggesting that such treatment reduced HRV disease and the inflammatory environment in malnourished hosts. Moreover, arachidonic acid is involved in the immune function of several organs and systems either directly or via conversion to eicosanoids (84, 85). Eiconoids exhibit a role in inflammation, protection of mucosal integrity of the gastrointestinal tract, and regulation of aggregation of platelets. In this study, TRP treatment increased levels of eicosanoids in PM samples indicating an enhancement of the immune function, inflammation, and maintenance of gut integrity. Cholesterol serves as the substrate for steroid hormone production and lower levels of cholesterol and is an indicator of protein-calorie malnutrition (86). In our current study, TRP (PL) and EcN + TRP (PM and FL) treatment groups had increased levels of cholesterol sulfate. This indicates that supplementation of EcN ± TRP has improved the nutritional status of malnourished pigs (15, 48). Gamma-butyrobetaine is an intermediate precursor of carnitine, and it promotes the development of functionally distinct microbial communities to optimize the metabolism of carnitine (87). This study shows that EcN + TRP treatment increased the intensities of gamma-butyrobetaine in FM analysis. Carnitine is an essential nutrient that plays a role in energy production and transport of activated fatty acids across the mitochondrial membrane during mitochondrial beta-oxidation, and its deficiency occurs in malnutrition (88). In this study, EcN treatment increased the levels of palmitoylcarnitine in FM and FL analysis. This indicates that EcN supplementation may have ameliorated malnutrition effects or total energy expenditure leading to increased survival rates and weight gain in malnourished piglets. Collectively, it suggests that there could be an important mechanism both locally and systemically for promoting diarrheal protection in EcN + TRP-treated pigs compared with untreated Def pigs.

### EcN ± TRP supplementation altered nucleotide-associated metabolites.

Cellular nucleotides, represented by purines and pyrimidines, play an important role in the synthesis of the nucleic acids RNA and DNA. Nucleotides play a vital role in host cell metabolism and are essential for viral replication dependent on the host supply of nucleotide biosynthesis. Many inhibitors targeting *de novo* nucleotide biosynthesis have been studied extensively in dengue virus, hepatitis B virus, hepatitis E virus, and other virus infection models (89–96). Purine metabolism plays a principal role in cellular redox regulation and accelerated tissue ATP degradation, and it is significantly reduced with malnutrition (97). EcN ± TRP treatments increased hypoxanthine levels in FM while EcN + TRP treatment also increased inosine and xanthine levels in PM. This suggests that the supplementations upregulated purine metabolism that coordinated the actions of salvage and *de novo* biosynthetic pathways improving the immune and growth functions. Cytidine-5′-monophosphate is a pyrimidine base found in RNA that helps to promote new cell growth. EcN treatment increased cytidine-5′-monophosphate levels in plasma indicating that the EcN-mediated increase in cytidine-5′-monophosphate synthesis may in part explain improved weight gain in the treated group relative to untreated Def pigs. NAD is an essential pyridine nucleotide that serves as a cofactor and substrate for several critical cellular processes involved in oxidative phosphorylation and ATP production, DNA repair, epigenetically modulated gene expression, intracellular calcium signaling, and immunological functions (98). NAD depletion may occur due to excessive DNA damage due to free radicals, energy crisis and reduced ATP production, chronic increase in immune activation, and inflammatory cytokines production (99). We observed that EcN supplementation to Def pigs substantially increased NAD levels in plasma compared to other treatments. This suggests that EcN may act via tryptophanase that can lead to *de novo* NAD synthesis in mammalian cells (100), which may have led to improved immune activation, barrier function, and anti-inflammatory response.

Low vaccine efficacy and environmental enteric dysfunction exacerbate HRV prevalence and severity in developing countries necessitating optimization of the existing and/or development of alternative preventive approaches. The combination of EcN + TRP or individual (EcN or TRP) treatments exhibits the potential to serve as a safe, natural, prophylactic treatment aimed at reducing HRV diarrhea in malnourished hosts, and it is accomplished at least in part via modification of the host lipid, bile acid, steroid hormone, and amino acid/peptide metabolism. In addition, EcN and TRP metabolism may have exerted direct antiviral activity against HRV by interfering with HRV infection, replication, and pathogenesis, improving gut barrier function and modulating mucosal immune responses, especially inflammatory processes that influence the development of HRV diarrhea. Our data on direct modulation of the host metabolism and immunity indicate that while EcN and TRP may provide effective therapeutics against HRV, this combination likely provides broadened and enhanced protection possibly against other enteric pathogens.

In summary, our results suggest that the EcN probiotic and TRP combination conferred greater protection in most cases (rather than individual EcN and TRP treatments). Moreover, EcN + TRP treatment (compared with other treatment groups) mostly upregulated immunoregulatory while downregulating proinflammatory cytokines following VirHRV infection in malnourished humanized Gn piglets. Our results suggest that low-cost dietary supplementation of combined EcN and TRP can potently ameliorate HRV-associated diarrhea and possibly other enteric infections.

## MATERIALS AND METHODS

### Human infant fecal microbiota.

The collection and use of HIFM were approved by The Ohio State University (OSU) Institutional Review Board. With parental consent, sequential fecal samples were collected from a healthy, 2-month-old, exclusively breastfed, vaginally delivered infant. Samples were pooled and diluted to 1:20 (wt/vol) in PBS solution containing 0.05% (vol/vol) cysteine and 30% glycerol and stored at −80°C as described previously (15, 26).

### Virus.

The Gn pig passaged virulent human rotavirus (VirHRV) Wa strain at pig passages 25 to 26 was used as a challenge virus at a dose of 1 × 10^6^ focus-forming unit (FFU) per pig as described previously (15, 26).

### Animal experimentation.

**FIG 6:**
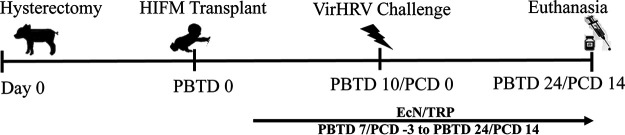
Experimental design showing time points for hysterectomy, human infant fecal microbiota (HIFM) transplantation, E. coli Nissle (EcN) 1917 probiotic/tryptophan (TRP) treatment, virulent human rotavirus (VirHRV) challenge, and euthanasia. PBTD, postbacterial transplantation day; PCD, PBTD; postchallenge.

All animal experiments were approved by the Institutional Animal Care and Use Committee at the OSU. Near-term pregnant sows (Landrace × Yorkshire × Duroc cross-bred) from the OSU swine center facility were used to derive the Gn pigs by cesarean section, and the Gn pigs were maintained in sterile isolators (101). Neonatal pigs obtained from five litters (5 to 15 pigs/litter) were randomly assigned to the following groups: (i) deficient (Def) diet (*n* = 6), (ii) deficient (Def + EcN) diet with EcN 1 × 10^5^ CFU/dose supplementation (*n* = 6), (iii) deficient (Def + TRP) diet with 0.4-g/day l-tryptophan (TRP) supplementation (*n* = 7), (iv) deficient (Def + EcN + TRP) diet with EcN and TRP combined supplementation (*n* = 7), and (v) sufficient (Suf) diet (*n* = 7). EcN and/or TRP supplementations were administered orally at 7 day following HIFM transplantation (Fig. 6). Deficient pigs were fed a diet of 50% Parmalat and 50% sterile water, which fell within NRC standards for calories, fat, and carbohydrates, while protein levels were half the recommended levels (7.5% versus 15% of diet). To confirm sterility from bacterial or fungal contamination before HIFM transplant, aerobic and anaerobic cultures of fecal swabs were performed. Additionally, all piglets were confirmed negative for the enteric viruses rotavirus, transmissible gastroenteritis virus, porcine epidemic diarrhea virus, calicivirus/sapovirus, astrovirus, and kobuvirus before HIFM inoculation. Four days postderivation, Gn pigs were inoculated orally with 2 ml of diluted HIFM stock, postbacterial transplantation day 0 (PBTD 0). Nine to 10 days were allowed for the establishment and stabilization of the fecal microbiota transplant. At PBTD 9 to 10, pigs were challenged with VirHRV (1 × 10^6^ FFU, postchallenge day/PCD 0). All pigs were humanly euthanized at PBTD 24/PCD 14. Blood, spleen, duodenum, and ileum were collected to isolate mononuclear cells (MNCs) for subsequent immunologic assays (33, 47, 102, 103). Serum, small intestinal contents (SIC), and large intestinal contents (LIC) were collected to determine the impact of EcN, TRP, and Def diet on VirHRV infection.

### Assessment of clinical signs.

Piglets were weighed and fecal swabs were collected post-VirHRV challenge. Fecal consistency was scored as follows: 0, normal; 1, pasty/semiliquid; and 2, liquid, and pigs with fecal scores more than 1 were considered diarrheic (10–12, 48, 58, 104, 105). Fecal swabs were suspended in 2 ml of minimum essential medium (MEM) (Life Technologies, Waltham, MA, USA), clarified by centrifugation at 800 × *g* for 10 min at 4°C, and stored at −20°C until quantification of infectious HRV by a cell culture immunofluorescence assay, as previously described (10–12, 48, 58, 104, 105).

### Isolation of mononuclear cells and immunological analyses.

Systemic (blood, spleen) and intestinal cells (duodenum, ileum) were collected to isolate MNCs as described previously (33, 47, 102, 103). The purified MNCs were suspended in E-RPMI 1640. The viability of MNCs preparation was determined by trypan blue exclusion (≥95%). The HRV-specific antibody-secreting cells (ASCs) and HRV-specific antibody titers in serum and intestinal contents were quantified as previously described (11, 15, 47, 58, 106, 107). Freshly isolated MNCs were stained for quantification of different T-cell subsets: T-helper cells (CD3^+^CD4^+^), cytotoxic T cells (CD3^+^CD8^+^), and T-regulatory cells (CD4/CD8^+^CD25^+^FOXP3^+^) (102, 105). Frequencies of IgA^+^ B lymphocytes were determined by identifying CD79β and IgA expression in MNCs as reported previously (58). MNCs were also stained to assess frequencies of plasmacytoid dendritic cells (pDCs) CD103^+^ (SWC3a^+^CD4^+^). Toll-like receptor (TLR) expression on MNCs was assessed using monoclonal antibodies to porcine and human cell surface markers as reported previously (10, 58, 59, 108). TLR4 (ligand bacterial lipopolysaccharide), TLR3 (ligand double-stranded RNAs), and TLR9 (ligand bacterial CpGs) were assessed in our experiments. Frequencies of natural killer (NK) cells (SWC3a^+^CD16^+^) were also assessed among systemic MNCs (105). Appropriate isotype-matched irrelevant antibodies were included as negative controls. Subsequently, 50,000 events were acquired per sample using BD Accuri C6 flow cytometer (BD Biosciences, San Jose, CA, USA). Data were analyzed using C6 flow sampler software. To analyze NK cytotoxicity, total blood MNCs and K562 cells were used as effector and target cells, respectively. For the effector, target cell ratios of 10:1, 5:1, 1:1, and 0.5:1 were used, and the assay was performed as described previously (105, 109). The Annexin V Apoptosis Detection kit APC (eBiosciences, San Diego, CA) and Propidium Iodide Staining Solution (eBiosciences) were used according to the manufacturer protocols to detect and discriminate apoptotic and necrotic MNCs. Within 4 h after the staining, acquisition of 50,000 events was performed using Accuri C6 flow cytometer, and analyses were performed using CFlow software (10, 38).

### Serum cytokines.

Serum samples were collected at the terminal time point and analyzed for proinflammatory (IL-1β, IL-6, TNF-α, IFN-α, and IFN-γ) and T-regulatory (IL-10 and TGF-β) cytokines as described previously with some modifications by increasing incubation timings of antibodies and samples (44, 102, 105).

### Metabolomics and lipidomics analyses.

Plasma and fecal samples were prepared using the cold methanol extraction method, clarified up with 0.2-μm ultracentrifugation spin filters, and further diluted with water containing 0.1% formic acid and subjected to nontargeted metabolic profiling. Briefly, samples were analyzed by ultrahigh performance liquid chromatography/tandem mass spectrometry (UHPLC/MS/MS) with Thermo LTQ Orbitrap XL (Thermofisher, CA). Metabolites were identified by automated comparison of the ion features in the experimental samples to a reference library of chemical standard entries that included retention time, the mass-to-charge ratio (*m/z*), preferred adducts, as well as associated MS spectra, and were quality controlled using pooled sample alignment using the Progenesis QI metabolomics package.

### Statistical analysis.

All statistical analyses were performed using GraphPad Prism version 6 (GraphPad Software, Inc., La Jolla, CA). Log_10_-transformed isotype ELISA antibody titers were analyzed using one-way ANOVA followed by Duncan’s multiple range test. Data represent the mean numbers of HRV-specific antibody-secreting cells per 5 × 10^5^ MNCs and are analyzed using a nonparametric *t* test (Mann-Whitney). The mean total fecal score was from PCD1 to PCD6. Mean fecal scores were analyzed by two-way ANOVA followed by Bonferroni posttest. Correlation analysis was performed using Spearman’s nonparametric correlation method. Significant differences were as follows: *, *P* < 0.05; **, *P* < 0.01; and ***, *P* < 0.001. Error bars indicated the standard error of the mean. Metabolomics data were analyzed with both the Progenesis software and XCMS Online from the Scripps Metlin Database. Data were normalized by sum and autoscaled. Heatmap visualization was performed based on the reorganization of metabolites to show the contrast between the groups. Principal-component analysis (PCA) was used for classification analyses. Treatment groups were compared with untreated pig groups and are presented as fold change.
